# Forensic trace DNA analysis to answer activity-level questions in a realistic-scenario activity study based upon the case of Idaho v Bryan C. Kohberger

**DOI:** 10.1016/j.fsisyn.2026.100690

**Published:** 2026-05-27

**Authors:** Aldrin Alviar, Ray Wickenheiser, Ashley Hall

**Affiliations:** a Forensic Science Graduate Program, Division of Continuing and Professional Education, University of California Davis, 1909 Galileo Ct. Ste B, Davis, CA, 95618, USA; b Ray Wickenheiser Forensic Consulting, Youngsville, LA, 70592, USA; c SupreMetric, LLC, Albany, NY, USA

## Abstract

An activity-level proposition is a hypothesis informed by case information about actions at a crime scene; it aids the fact-finder in addressing the “how” and “when” of the evidence, not just the “who.” Activity-level reporting focuses on probabilities of the findings [or results] from crime scene evidence given alternate propositions. Empirical data generated by simulating real-life activity scenarios can be of great value to the forensic scientist in their evaluations of crime scene evidence. In this study, experiments were designed to approximate circumstances relevant to the case of the State of Idaho vs Bryan C. Kohberger, whose DNA was found on a knife sheath at the apartment where four University of Idaho students were stabbed to death. Two competing activity-level propositions concerning the origin of the DNA on the knife sheath were examined: 1) Kohberger touched the snap (direct DNA transfer), or 2) Kohberger, the POI, shook hands with another person who touched the snap (indirect DNA transfer). Empirical data were generated by simulating both activity pathways independently, quantifying DNA recovery and generating DNA profiles to inform the probabilities used to evaluate the findings given the competing activity-level propositions. Significantly greater quantities of POI DNA were recovered following direct transfer, and major POI DNA profiles were more likely if a direct transfer occurred rather than an indirect transfer.

## Highlights

• Activity-level reporting is necessary for jury understanding of the *why* and *how*.
• Domesticated fingerprint model enables controlled trace DNA transfer studies.
• Experiments compare direct and indirect DNA transfer to a knife sheath snap.
• Direct transfer yields significantly greater POI DNA recovery than indirect.
• Indirect transfer typically produces mixtures dominated by the last handler.

## 1 Introduction

An activity-level proposition is a hypothesis that is informed by case information when evaluations of evidence given the actions at a crime scene are of interest; it helps the fact-finder to address the “how” and the “when” the evidence arrived at the crime scene. For example, consider a hypothetical scenario: your DNA is found on a knife handle at the scene of a stabbing. One side presents this as evidence that you stabbed the victim with the knife. The other side counters by positing that you shook hands with the actual perpetrator, who stabbed the victim with the knife. If it cannot be reasonably disputed that it is your DNA on the knife, then the critical question becomes “how did the DNA get there?”

To understand activity-level propositions, it is important to be aware of the distinctions captured by different hypotheses of a crime, which are best described as a “hierarchy of propositions.” The hierarchy comprises the different levels at which questions can be asked and evidence can be interpreted, including the sub-sub-source (what is the databasescellular origin or tissue source of DNA), sub-source (who is the source of the DNA?), the source (who is the source of a specific sample, e.g. the donor of a semen stain?), the activity (how or when was the DNA deposited?) and the offense level (if the activity is or is not a crime) [1,2].

The step from determination of sub-sub-source, sub-source or source to activity-level may seem trivial when only one reasonable explanation for DNA transfer has been offered, but it is not straightforward if a plausible alternative has been presented, as in the hypothetical stabbing described earlier. If only evaluations given sub-source or source propositions are provided, the trier-of-fact, typically a jury, is left to weigh the findings in the context of the case [3], but with no knowledge to do so. The forensic scientist, however, is better-equipped to address activity-level, particularly when trace levels of DNA are present as considerations of transfer, persistence, prevalence and recovery (TPPR) require specialized forensic knowledge [4].

The effects of factors such as TPPR can be evaluated by means of a likelihood ratio (LR), which is ideally populated using empirical data from the literature or produced experimentally in-house [[5], [6], [7]] prior to any knowledge of technical findings in the case under evaluation. The LR is comprised of a pair of mutually exclusive propositions, expressed as the ratio of two conditional probabilities: 1) the probability of the findings if one proposition is true given the conditioning information and 2) the probability of the findings if the other proposition is true given the conditioning information [7,8]. The LR is a measure of the relative strength of support that scientific findings provide for one proposition versus another; it is always focused on the findings, never on the propositions [8].

Forensic practitioners surveyed in the U.S. and internationally generally support the use of activity-level, or evaluative reporting, but cite limitations including the lack of sufficient empirical data generated from realistic-scenario activity studies [9,10], and the limited availability of statistical databases, e.g containing quantitative measurements of DNA recovery, transfer frequencies, persistence outcomes, profile quality metrics, substrate effects, transfer mechanisms (direct versus indirect), environmental influences, or probabilities associated with observing particular DNA profile outcomes under specified activity propositions, that would be useful in informing the conditional probabilities captured in likelihood ratios [11]. While there have been a number of peer-reviewed activity studies evaluating the transfer of trace DNA in various scenarios [[12], [13], [14], [15]], a limitation lies in the inter- and intra-person variability in the DNA content of trace samples. The majority of the studies have included semi-quantitative variables; the absolute quantity of DNA in the starting material, e.g. a fingerprint or palm shedding, was not known and results were evaluated relative to other results in the same study. These studies have demonstrated that DNA can be transferred through both passive transfer, in which DNA is deposited unintentionally through routine contact or handling, and active transfer, in which deliberate or forceful interaction facilitates DNA deposition, as well as through direct and indirect contact [[16], [17], [18], [19], [20], [21], [22]]. The transfer is significantly affected by factors such as the nature and number of surfaces and/or donors [[23], [24], [25], [26]], the pressure and friction of the touch [[20], [21], [22],^25^,^27^], donor propensity to shed cells [28], and the persistence of DNA [29,30].

In the work described here, we used a ground truth, trace DNA positive control, the domesticated fingerprint (DFP). The DFP is a simulated fingerprint that contains a known quantity of DNA in a background of sebaceous fingerprint chemistry [31], eliminating the human shedder variable [28,32,33]. Incorporating the ground truth DFP into transfer experiments permits absolute quantification of DNA recovery. The DFP is the lab-created version of the wild fingerprint, the sample naturally deposited by human hands. Using a defined starting amount of DNA enabled quantification of DNA recovery and loss across transfer pathways, yielding empirical data that may be useful in informing the probability values used in likelihood ratio assignment for activity-level evaluations.

In the realistic-scenario activity study, we conducted experiments designed to approximate circumstances relevant to the case of the State of Idaho v Bryan C. Kohberger. Specifically, the study modeled competing direct and indirect DNA transfer pathways associated with DNA recovered from a knife sheath snap. We present quantitative data and DNA profiling results from both direct and indirect DNA transfers. DNA profiling data is used to generate transfer probability tables, similar to the approach described by Samie et al. [34] These data may serve as reference tables for actual casework, demonstrating the utility of performing bespoke experiments of relatively achievable sample sizes that laboratories could complete to generate a library of probability values to use in informing activity level likelihood ratios. Finally, we discuss six emerging theories of trace DNA evidence supported by this empirical data.

## 2 Methods

All human samples were collected from donors with informed consent with the approval of the Institutional Review Board at the University of California, Davis (Protocol 1956436). All methods were carried out in accordance with relevant guidelines and regulations.

### 2.1 Sebaceous fingerprint solution

The sebaceous fingerprint solution was made in two parts [26,31]. The first part contained eccrine components in 100 ml sterile H_2_O: 140 mg potassium chloride (Fisher Scientific, Waltham MA); 130 mg sodium chloride (Fisher Scientific); 25 mg sodium bicarbonate (Fisher Scientific); 17.5 mg ammonium hydroxide (Fisher Scientific); 4.0 mg magnesium chloride (Sigma-Aldrich, St. Louis, MO); 27.5 g serine (Sigma-Aldrich); 13.5 g glycine (Sigma-Aldrich); 11 g ornithine (Sigma-Aldrich); 8 g alanine (Sigma-Aldrich); 4 g aspartic acid (Sigma-Aldrich); 4 g threonine (Sigma-Aldrich); 4 g histidine (Sigma-Aldrich); 3 g valine (Sigma-Aldrich); 3 g leucine (Sigma-Aldrich); 190 g lactic acid (Sigma-Aldrich); 50 g urea (Sigma-Aldrich); 2 g pyruvic acid (Sigma-Aldrich); 0.5 g acetic acid (Fisher Scientific); and 0.5 g hexanoic acid (Sigma-Aldrich). To make the eccrine solution: 1) all the chemicals were combined in 90 ml sterile water; 2) sonicated in water bath for 15 min; 3) pH was adjusted to 5.5 with 5M NaOH (Fisher Scientific); 4) volume was adjusted to 100 ml; 5) solution was autoclaved at 121 °C and 15 psi.

The second part, containing sebaceous components, was combined in 20 ml sterile H_2_O: 50 mg hexanoic acid (Sigma-Aldrich); 50 mg heptanoic acid (Sigma-Aldrich); 50 mg octanoic acid (Sigma-Aldrich); 50 mg nonanoic acid (Sigma-Aldrich); 50 mg dodecanoic acid (Sigma-Aldrich); 50 mg tridecanoic acid (Sigma-Aldrich); 50 mg myristic acid (Fisher Scientific); 50 mg pentadecanoic acid; (Sigma-Aldrich) 55 mg palmitic acid (Fisher Scientific); 55 mg stearic acid (Fisher Scientific); 50 mg arachidic acid (Sigma-Aldrich); 55 mg linoleic acid (Sigma-Aldrich); 55 mg oleic acid (Sigma-Aldrich); 275 mg triolein (glyceryl triolate) (Sigma-Aldrich); 20 mg tricaprylin (trioctanoin) (Fisher Scientific); 20 mg tricaprin (Sigma-Aldrich); 20 mg trilaurin (Sigma-Aldrich); 20 mg trimystrin (Fisher Scientific); 20 mg tripalmitin (Fisher Scientific); 120 mg squalene (Fisher Scientific); 30 mg cholesterol (Fisher Scientific); 40 mg Cholesterol n-decanoate (Santa Cruz Biotechnology, Dallas, TX); 155 mg cetyl palmitate (Fisher Scientific). To make the solution: 1) sebaceous components were added to an amber bottle and mixed the on a hot plate at 35 °C using the magnetic stirrer; 2) equal amounts of the sebaceous mixture and the eccrine mixture were added in to a separate bottle; 3) 1 ml BRIJS20 (0.05 mg/ml) (Sigma-Aldrich) was added; 4) the final solution was mixed on a hot plate at 35 °C with the magnetic stirrer. The solution was not autoclaved.

To make BRIJ, 1 mg BRIJ S20 was dissolved in 20 ml of sterile water using magnetic stirrer and hot plate at 35 °C.

### 2.2 Cell suspension

Buccal epithelial cells were collected by swabbing the donor’s interior cheek using a sterile cotton-tipped swab (Puritan, Guilford, ME). The cotton tip was separated from the shaft and was placed into a 1.5 mL microcentrifuge tube with 500 μL of 1X Accumax™ Cell Dissociation Solution (Innovative Cell Technologies, San Diego, CA) for a 15-min incubation at room temperature, with gentle vortexing every 5 min for 10 s. The swab was then removed from the tube and an additional 500 μL of Accumax™ was added followed by another 15-min incubation at room temperature with gentle vortexing every 5 min for 10 s.

### 2.3 LUNA™Automated cell counter

Diamond™ Nucleic Acid Dye (Promega, Madison, WI) was diluted 1:10^4^ in sterile water. A 1:1 dilution of cell suspension:dye was prepared. Ten microliters of the mixture was pipetted onto LUNA™ Reusable Slide (Logos Biosystems, Annandale, VA) and placed into the LUNA-FL™ Dual Fluorescence cell counter (Logos Biosystems). Cells were counted using the fluorescence cell counting mode. The operating parameters were Dilution 2; Minimum cell size 5; Maximum cell size 60; green fluorescence threshold 7; and red fluorescence threshold 7. The tag option was used to assure each cell was counted. Three separate 10 μL aliquots from a single suspension were counted three times each, resulting in a total of 9 data points. Mean cell count was calculated to obtain the concentration of the cell suspension (cell/μL).

### 2.4 Domesticated fingerprints (DFPs)

The mean number of cells per microliter of suspension was converted to the volume of suspension required to deliver a specific quantity of DNA (1 epithelial cell = 6 pg DNA). The volume was brought to 18 μL in 1X PBS and combined with 2 μL of 10X sebaceous fingerprint solution. To deposit a DFP, the entire 20 μL was pipetted onto a surface. To collect, a sterile cotton-tipped swab (Puritan, Pittsfield ME) was wetted with 20 μL 2% SDS and the entire fingerprint was collected.

### 2.5 Wild fingerprints (WFPs)

To deposit a wild (true) fingerprint, donors were asked to refrain from washing their hands for at last 1 h before their donation. To deposit a sample, they were asked to contact a surface with firm pressure for 10 s. To collect, a sterile cotton-tipped swab (Puritan) was wetted with 20 μL 2% SDS and the entire fingerprint was collected.

### 2.6 Domesticated hand

**Fig. 1:**
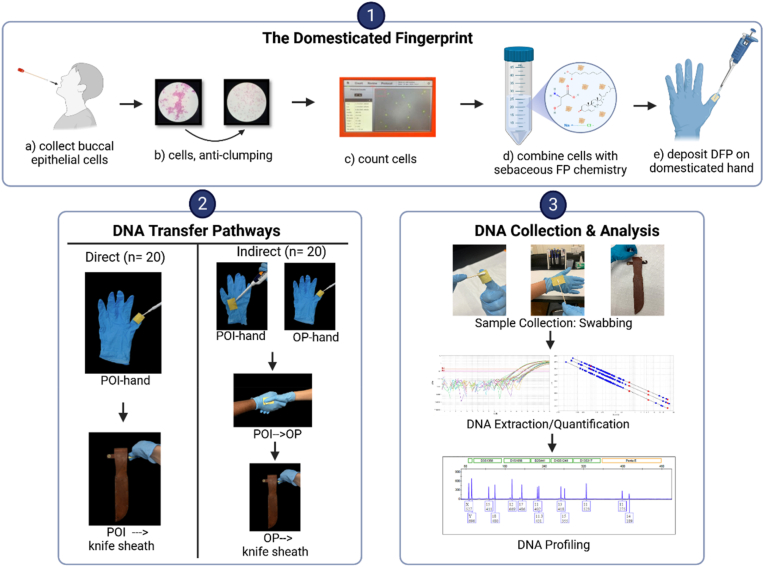
Realistic-Scenario Activity Studies, Direct v Indirect Transfer. 1) The domesticated fingerprint is produced and deposited in a four-step process; 2) To simulateDirect and Indirect DNA transfers using DFPS deposited on domesticated hands. ; 3) Samples are collected from each hand and the snap by swabbing, the DNA is extracted, quantified by real-time PCR and a DNA profile is generated. (*Created in BioRender. Hall, A (2026)*https://BioRender.com/c8dub7y, chemical structures from PubChem, https://pubchem.ncbi.nlm.nih.gov/:https://pubchem.ncbi.nlm.nih.gov/compound/5997, https://pubchem.ncbi.nlm.nih.gov/compound/Octanoic-Acid, https://pubchem.ncbi.nlm.nih.gov/compound/5951, https://pubchem.ncbi.nlm.nih.gov/compound/5234).

The domesticated hand was constructed using a nitrile glove (ThermoFisher Scientific, Waltham MA) and an epidermal skin equivalent, Lorica Leather® [35,36](Ehrlich Leder, Biberach Germany). The leather was affixed to the nitrile glove with Gorilla Glue (Gorilla Glue Company, Cincinnati OH). To initiate a transfer, a 20 μl domesticated fingerprint was pipetted onto the Lorica Leather® then transferred to the next surface (please see Fig. 1 for a visual depiction of this process).

### 2.7 Surface decontamination

The brown leather knife sheath fit a KA-BAR 1217S with a 7’ blade, had a USMC Logo, and was purchased online (Amazon, https://www.amazon.com/KA-BAR-1217S-Leather-Sheath-Knife/dp/B000MAZIA0/). The knife sheath and Lorica Leather® “skin” were decontaminated before use to ensure no background DNA remained on the surfaces. The surfaces were subjected to UV using the UV Stratalinker 1800 (Stratagene, San Diego, CA) for 30 min. Following UV treatment, DNA Away (ThermoFisher Scientific) was pipetted over the entire surface allowed to dry for 10 min. A cotton swab wetted with DNA Away solution was used to swab the surface with moderate pressure, followed by another swabbing using a cotton swab wetted with 70% ethanol. To confirm that each surface was DNA-free, a sterile, cotton-tipped swab (Puritan) wetted with 20 μL of 2% SDS was used to swab each surface, snap and hand(s). These negative controls were included in each sample set and analyzed with fingerprint samples to confirm the absence of background DNA.

### 2.8 DNA extraction

DNA was extracted using the DNA IQ™ System (Promega). The manufacturer’s protocol was optimized for use with trace DNA samples. Briefly, a swab was added to a DNA IQ™ Spin Basket (Promega) and placed in a in a ClickFit Microtube (Promega). Four hundred microliters of 1X Lysis Buffer/4 μL 1 M DTT was added, and the samples were incubated overnight (12 – 18 h) at 70 °C. After incubation, the tubes were centrifuged 16.3xg in a Spectrafuge 24D benchtop centrifuge (Labnet, Edison NJ) for 2 min to remove the excess liquid from the swab to the lysate in the tube. Seven microliters of DNA IQ ™ Resin were added to each tube and they were placed in a MagneSphere® Technology Magnetic Separation Stand (Promega). The supernatant was removed and discarded. One hundred microliters of the Lysis Buffer/DTT mix were added. The tube was vortexed briefly, placed in the magnetic stand, and the supernatant was removed. One hundred microliters of the 1X Wash Buffer were added. The tube was vortexed and placed back in the magnetic rack, and the supernatant removed. This was repeated an additional two times, for a total of three washes. After the final wash, the supernatant was removed, and the pellet was dried at room temperature for 5 min. In the final step, DNA was eluted by incubation at 65 °C in 30 μL Elution Buffer for 5 min. The eluate was removed to a clean, sterile tube and stored at 4 °C until use.

### 2.9 DNA quantification: Quantifiler™ Trio DNA Quantification Kit

DNA was quantified using the Quantifiler™ Trio DNA Quantification Kit (ThermoFisher Scientific) according to the manufacturer’s protocol. Briefly, reactions were prepared by combining 2 μL of purified DNA, 8 μLQuantifiler™ Trio primer mix, and 10 μL Quantifiler™ THP PCR reaction mix in a total volume of 20 μL. A five-point standard curve ranging from 0.005 to 50 ng/μL was made using Quantifiler™ THP DNA Standard diluted using Quantifiler™ THP Dilution buffer. Dye settings, threshold and baseline settings were followed according to the manufacturer’s protocol. Cycling conditions were: 1) initial denaturation of 95 °C for 2 min, 2) 40 cycles of denaturation at 95 °C for 9 s and annealing/extension at 60 °C for 30 s. Total human nuclear DNA was quantified using the values from the small autosomal marker. Male DNA was quantified using values from the Y-marker. The quantity of female DNA was estimated as [small autosomal marker] – [Y-marker].

### 2.10 PCR amplification and genetic analysis

Forty total DNA samples were amplified using the PowerPlex® Fusion System Amplification Kit (Promega), according to the manufacturer’s protocol, with 1.0 ng DNA added to each reaction. If the DNA extract concentration was insufficient to add 1.0 ng of template DNA to the PCR, the maximum allowable volume of 15 μL was added PCR products were analyzed using the SeqStudio Genetic Analyzer (ThermoFisher Scientific). One microliter of PCR product was mixed with 0.5 μL of Promega ILS 500 Lane standard and 9.5 μL of Hi-Di™ Formamide (ThermoFisher Scientific). Samples were electrophoresed on the SeqStudio Genetic Analyzer with the following run parameters: Promega 5C dye set; Capillary temperature: 60 °C; Pre-run voltage: 13000V; Pre-run time: 180s; Injection voltage: 1200V; Injection time: 7s; Run voltage: 9000V; Run ramp duration: 300s; Run time: 1440s. The DNA profile was analyzed using GeneMapper™ ID-X Version 1.6.

### 2.11 Mixture analysis

The standardized method used for calculating male and female contributions in mixed DNA samples using STR peak height data is based on relative fluorescent unit (rfu) measurements from electropherogram data. The Amelogenin locus was omitted from the analysis, and an analytical threshold of 110 rfu was set. Alleles were classified as male, female, shared (present in both), or unknown by comparison of the reference and mixed DNA profiles generated from the fingerprint samples. Male and female rfu contributions were summed across all loci. The total mixture ratio was expressed as: Male % = (Σ Male RFU/Σ Total RFU) × 100; and Female % = (Σ Female RFU/Σ Total RFU) × 100. For shared alleles, the rfu value was divided equally (50/50) between male and female. Results were reported as overall male and female mixture percentages.

### 2.12 Activity methodology

For the direct transfer pathway (Proposition 1: POI-hand → knife sheath snap), DNA from a single male donor was deposited onto the POI-hand as a 9.0 ng DFP. The POI-thumb immediately contacted the knife sheath snap for 10 s using firm pressure. DFPs were immediately sampled and/or transferred following deposition in order to better approximate the moist eccrine and sebaceous environment associated with naturally deposited fingerprints and touch DNA residues. Following completion of the single-step transfer event, samples were collected from both the POI-thumb and the knife sheath snap. This direct transfer experiment was repeated independently twenty times using freshly decontaminated hands and knife sheath snaps for each trial. DNA was extracted from all samples and quantified using real-time PCR.

The same transfer was repeated twenty times using non-gloved wild hands. The POI thumb contacted the knife sheath snap for 10 s with firm pressure. A sample was collected from the knife sheath snap.

For the indirect transfer pathway (Proposition 2: POI-hand → OP-hand → knife sheath snap), DNA from two contributors was used to model a more realistic transfer scenario in which both hands contributed DNA and bidirectional transfer could occur during contact. A 9.0 ng male POI-DFP was applied to the POI-hand (dorsal web space between the index finger and thumb), and a 6.0 ng female OP-DFP was applied to the OP-hand (thumb). The POI-hand and OP-hand engaged in a handshake for 10 s using firm pressure, after which the OP-thumb contacted the knife sheath snap for 10 s with firm pressure (Fig. 1). DFPs were immediately sampled and/or transferred following deposition in order to better approximate the moist eccrine and sebaceous environment associated with naturally deposited fingerprints and touch DNA residues. Following completion of the multi-step transfer pathway, samples were collected from all three surfaces to monitor DNA transfer and persistence: (1) the POI-hand (dorsal web space between the index finger and thumb), (2) the OP-hand (thumb), and (3) the knife sheath snap. This pathway was independently repeated twenty times using freshly decontaminated hands and snaps for each trial.

The same transfer was repeated twenty times for wild fingerprints, using ungloved hands. Samples were collected from: 1) POI hand (dorsal web space between the index finger and thumb); 2) OP hand (thumb); 3) knife sheath snap.

## 3 Results and discussion

### 3.1 DNA transfer in the State of Idaho vs. Bryan C. Kohberger

To conduct a realistic-scenario activity study and generate empirical data that may contribute to the growing body of transfer research available to the forensic science community, we mocked a true crime scene - the case of the State of Idaho v Bryan C. Kohberger. Mock reconstructions of crime scenes are inherently approximations and are subject to limitations in available information, environmental fidelity, and technical constraints. However, efforts are made to maximize realism and maintain experimental relevance within these constraints. In this case, we know that, on November 23, 2022, four University of Idaho students - Madison Mogen, 21; Kaylee Goncalves, 21; Xana Kernodle, 20; and Ethan Chapin, 20 - were stabbed to death in their off-campus residence in Moscow, Idaho. DNA matching Kohberger was found on a knife sheath at the scene of the crime. For convenience, we referred to the alleged perpetrator as the person of interest (POI), and a second, unnamed person as the “other person” (OP). In casework, propositions are typically developed with consideration of case circumstances, available information, and relevant expertise, often in consultation with prosecution and/or defense; in the absence of such consultation here, the propositions are necessarily simplified and may not encompass all plausible transfer scenarios.

We offered two alternate explanations for the DNA at the scene: Proposition 1) Kohberger, the POI, touched the knife sheath (direct transfer); or Proposition 2) Kohberger shook hands with some other person (OP) who touched the knife sheath (indirect transfer). The latter is not the only indirect pathway by which the DNA could have arrived on the knife sheath, but it is the most reasonable indirect pathway by which we expect the most DNA would be deposited, therefore the most conservative estimate of the activities. In this study, ‘conservative’ refers to analytical choices that were intended to avoid over-attribution of DNA to a particular contributor or transfer mechanism, thereby reducing the likelihood of overstating the strength of the observed findings. Additionally, although the POI could have touched other surfaces, such as the leather of the sheath, we transferred the DFP to the surface we expected to yield the best results, the snap. Fig. 1 is an overview of the experimental design, from domesticated fingerprint (panel 1), to the direct and indirect pathways (panel 2), and finally DNA collection and analysis (panel 3).

### 3.2 Domesticated fingerprints with domesticated hand

To complete the Proposition 1 direct transfer, 9.0 ng male POI-DNA was placed on the thumb of the domesticated hand, constructed of an epidermal skin equivalent affixed to a nitrile glove (see Methods Section 2.6 for additional detail), which applied firm pressure to the snap for 10 s. The transfer was repeated 20 times, with an average of 0.41 ng DNA (SD = 0.08) or 4.5% of the DNA originally deposited, remaining on the POI-hand. We recovered a mean of 0.72 ng DNA (SD = 0.10) from the snap, a loss of 80% of the DNA initially deposited. Although only POI DNA was added to the direct transfer, low-level female DNA was detected on the POI hand (mean 0.065 ng, SD = 0.063) and on the snap (0.020 ng, SD = 0.045). In our experience, it is not unusual to detect low-level, non-self DNA in buccal samples, and this was the source of the female DNA in these samples.

Proposition 2 indirect transfers required the addition of DNA from two donors, the POI and the OP. In such cases, we need to account for the shedding propensity of each individual. For a conservative estimate of POI DNA transfer in a mixed-DNA sample, the POI, not the last handler (OP), would be the better shedder, donating more DNA to the pathway. A higher-shedding POI was simulated by adding 9.0 ng male DNA to the POI-hand (dorsal web space between the index finger and thumb)and only 6.0 ng female DNA to the OP-hand (thumb). The POI hand shook the OP hand for 10 s with firm pressure. The OP-hand pressed the knife sheath snap for 10 s with firm pressure to complete the transfer. The indirect transfer was repeated 20 times, and the DNA recovery averaged.

An average of 7.4% of the POI DNA deposited (0.67 ng, SD 0.21) and a mean 0.83% of the initial OP DNA (0.05 ng) were recovered from the POI-hand, showing low-level back-transfer (OP to POI) in this experimental pathway, and confirming that the majority of the POI DNA was transferred. From the OP-hand, we recovered a mean 8.3% of the added POI DNA (0.75 ng, SD 0.19) and an average 20% of OP DNA (0.61 ng, an indirect measurement estimated as [(small autosomal marker) – (Y-marker)], SD was calculated only for the direct measurement. From the final surface of the indirect transfer - the knife sheath snap - we recovered a mean 2.2% of the POI DNA that was added initially (0.20 ng, SD 0.09) and an average 5.0% of the OP DNA originally included (0.30 ng, an indirect measurement for which SD was not calculated). Welch’s *t*-test (t = 17.362, df = 38.431, p < 2.2 x 10^−16^) showed that significantly more POI DNA was recovered from direct than from indirect transfer. The data from Propositions 1 & 2 are summarized in Table 1. It is noteworthy that 1.5X more POI DNA (9.0 ng) than OP DNA (6.0 ng) was added to the indirect transfer experiment, but the mean DNA recovered from the snap contained 1.5X more OP DNA (0.30 ng) than POI DNA (0.20 ng). While the direct reversal of donor contributions may be a coincidence in this case, it is significant to note the dynamics of the transfer.

**Table 1:** DNA Recovery: Domesticated Fingerprints. Direct transfer: a 9.0 ng POI DFP was placed on the POI-Hand and immediately transferred to the snap; the OP-Hand was not used. Indirect pathway: a 9.0 ng POI DFP was placed on the POI-Hand, and a 6.0 ng OP DFP was placed on the OP-Hand. The POI hand shook the OP hand, which immediately pressed the knife sheath snap. The pathways were repeated independently twenty times each. The mean DNA recovery for each surface is listed and the standard deviation indicated. The number is parentheses is the percentage of the total DNA deposited that was recovered from that surface. The “OP DNA” is an indirect estimation [(total human DNA) – (Y-chromosomal DNA)], for which SD is not calculated.

DFP	Direct Transfer	Indirect Transfer		
	POI DNA (9.0 ng deposited)	POI DNA (9.0 ng deposited)	OP DNA (6.0 ng deposited)	Total DNA (15 ng deposited)
POI-Hand	0.41 ng, SD 0.08 (4.6%)	0.67 ng, SD 0.21 (7.4%)	0.050 ng (0.83%)	0.72 ng, SD 0.16 (4.8%)
OP-Hand	N/A	0.75 ng, SD 0.19 (8.3%)	0.61 ng (10.1%)	1.4 ng, SD 0.46 (9.3%)
Knife Sheath Snap	0.72 ng, SD 0.10 (8.0%)	0.20 ng, SD 0.09 (2.2%)	0.30 ng (5.0%)	0.50 ng, SD 0.19 (3.3%)

The data generated from the indirect transfers gave rise to four trace DNA Theories. First, the quantity of POI DNA transferred to the final surface was reduced by the contribution of the second individual. OP DNA (0.30 ng, 5.0% of the female DNA originally deposited) was recovered from the knife sheath snap after the indirect transfer; the quantity of POI DNA transferred was reduced by the contribution of the second individual and a new equilibrium established between the DNAs on each surface (Trace DNA Theory #1), which can be reflected in the subsequent transfer. After the indirect transfer, the combined total DNA recovered from the OP-hand and snap was 0.91 ng OP (0.61 ng OP-hand plus 0.30 ng snap) and 0.95 ng POI (0.75 ng OP-hand plus 0.20 ng snap). The mixture ratio from the OP-hand was 1:0.81 (M:F). The new equilibrium established on the snap resulted in a M:F ratio of 1:1.5. Similar results were seen across our study, where new DNA was added, a new mixture equilibrium was established.

Next, the mean POI DNA transferred by the indirect route was only 28% of the quantity transferred by the direct route. DNA is lost at each step of a multi-step transfer. POI DNA recovered from the OP-hand was 0.75 ng, while the POI DNA recovered from the snap was only 0.2 ng, showing diminishing DNA returns at each step (Trace DNA Theory #2), and with the majority contributor, in the majority of cases, the OP final handler (Trace DNA Theory #3). Throughout our study, we saw a continuum of losses of DNA in transfer steps, which include processing and analysis of DNA, reinforcing our theories of diminishing returns. In this case, the quantities of POI and OP DNA deposited were similar and further experimentation is needed to determine if this theory is universal. Results may differ if, for example, POI DNA is blood (high source) and OP is a fingerprint (low source).

### 3.3 Wild fingerprints with wild hands

The ultimate goal in developing the domesticated protocol was to provide a conservative estimate of the DNA loss/recovery resulting from DNA transfer in wild fingerprints. For comparison, we repeated the Idaho murders direct (Proposition 1) and indirect pathways (Proposition 2) with wild fingerprints and hands. A total of three male participants contributed to these experiments. Following each replicate pathway, participants waited at least 1 h without hand washing before contributing again. For the purposes of data analysis, all male DNA was assumed to originate from the POI and all female DNA from the OP. For Proposition 1, a male POI thumb firmly pressed on the snap of the knife sheath for 10s in a direct transfer. The transfer was repeated twenty times; the mean DNA recovered was 0.61 ng (SD = 0.57 range 0.047 – 1.87 ng, Table 2).

**Table 2:** DNA Recovery: Wild Fingerprints. For the direct pathway, a male POI-hand pressed the knife sheath snap. For the indirect pathway, a POI Dom-hand shook an OP-hand. The OP-hand immediately pressed the knife sheath snap. Each pathway was repeated independently twenty times. The mean DNA recovery for each surface is listed and the standard deviation indicated. The OP DNA is estimated as [(small autosomal marker) – (Y-marker)]; no SD was determined for this estimated value.

WFP	Direct Transfer	Indirect Transfer		
	POI DNA	POI DNA	OP DNA	Total DNA
POI-Hand	N/A	2.9 ng (SD 2.45)	0.48 ng	3.4 ng (SD 2.7)
OP-Hand	N/A	0.32 ng (SD 0.21)	4.5 ng	4.8 ng (SD 3.5)
Knife Sheath Snap	0.61 ng (SD 0.57)	0.055 ng (SD 0.086)	0.50 ng	0.56 ng (SD 0.71)

For Proposition 2, a male (POI) and a female (OP) shook hands for 10 s with firm pressure. The OP firmly pressed on the snap for an additional 10 s. We collected samples from: 1) male POI hand (dorsal web space between the index finger and thumb), 2) female OP hand (thumb), and 3) knife sheath snap. The pathway was repeated twenty times, and the recovery values averaged (Table 2). From the first hand in the pathway, the male POI, we collected an average of 2.9 ng POI DNA (SD = 2.5, range 0.56– 8.6 ng). We observed transfer from the OP to the POI hand as well, with 0.48 ng OP DNA collected from the male hand (range 0.0 – 2.9 ng); this was an indirect estimation ([small autosomal marker] – [Y-marker] for which no SD was calculated). The mean DNA collected from the second hand, the female OP, was: 0.32 ng male POI (SD = 0.21, range 0.027– 0.71 ng) and 4.5 ng female OP, (0.54 – 12. ng). From the knife sheath snap, the final step in the indirect transfer pathway, we collected 0.055 ng male POI DNA (SD = 0.086, range 0.00 – 0.28 ng) and 0.50 ng female OP DNA (0.0 – 2.4 ng). After the indirect transfer, the combined total DNA recovered from the OP-hand and snap was 5.0 ng OP (4.5 ng OP-hand plus 0.50 ng snap) and 0.38 ng POI (0.32 ng OP-hand plus 0.055 ng snap). The mixture ratio from the OP-hand was 0.070:1 (M:F). The new equilibrium established on the snap resulted in a M:F ratio of 0.11:1. With the addition of new DNA, a new mixture equilibrium was established, in agreement with the DFP results. Statistical analyses of the raw quantification data showed that significantly more male POI DNA was recovered from the knife sheath snap after the direct pathway than after the indirect pathway (Welch’s *t*-test, t = 4.2875, df = 19.881, p = 0.0003635).

In the wild pathways, the mean male POI DNA transferred to the snap by the indirect route was 10.8% of the quantity transferred by the direct route. However, the large SD values, roughly equal to or greater than the mean, complicate the interpretation. They demonstrate the great intra-personal variation in the DNA contents of a trace sample, highlighting the problems with the use of wild fingerprints in experiments designed to generate empirical data and show the value of the domesticated approach for replicates that look at specific steps of the overall activity and analysis process.

### 3.4 DNA profiling

DNA quantification experiments confirmed that there were significant differences in the quantity of POI DNA recovered from the final surface, the knife sheath snap, which were dependent upon the method of transfer, direct or indirect. However, in case work, the critical factor is the DNA profile. The original wild fingerprint DNA extracts (direct and indirect transfers) and domesticated fingerprint extracts (direct transfers) were lost due to a cold storage issue; an additional ten pathways were completed for each; extracts from the knife sheath snaps were used for DNA profiling. The peak heights (RFU) of the ten DNA profiles are presented in Table 3, Table 4. Quantification results for each sample are also provided, along with the quantity of DNA added to PCR.

**Table 3:** **DNA Profiles from DFP Direct Transfers.** For each of the ten replicate profiles generated from DNA collected from the knife sheath snaps, peak heights (RFU) are presented. DNA quantification results and the amount of DNA added to each PCR are also provided. An empty box indicates that no allele above the analytical threshold of 110 rfu was detected.

DFP: Direct	DD1	DD2	DD3	DD4	DD5	DD6	DD7	DD8	DD9	DD10
Quant (ng/μl)	0.0062	0.0060	0.012	0.035	0.025	0.028	0.043	0.022	0.031	0.057
Total DNA (ng)	0.18	0.18	0.37	1.1	0.75	0.83	1.3	0.65	0.92	1.7
Added to PCR (ng)	0.092	0.091	0.18	0.50	0.38	0.41	0.50	0.32	0.46	0.50

	rfu	rfu	rfu	rfu	rfu	rfu	rfu	rfu	rfu	rfu
Amel	149	241	111	644	683	555	1216	635	1416	1000
		191	353	585	431	492	1251	622	1299	1562
D3	233		194	897	613	657	1456	596	938	1344
	244		122	680	801	555	996	409	1044	1199
D1		191	289	509	359	785	1193	326	1664	1261
		173	224	610	344	579	955	465	1016	817
D2S441			117	388	207	402	710	208	883	452
			140	362	445	454	625	236	789	833
D10			153	348	276	331	548	186	726	971
				215	380	363	621	274	1069	855
D13			233	406	447	362	796	343	1797	1428
PentaE					169	148	342		437	304
							132		307	373
D16	172		194	271	301	289	563	356	913	372
	138		120	431	278	330	689	275	948	678
D18				183	242	203	410	147	443	284
					227		312		277	235
D2		134		387	686	439	717	247	575	504
CSF				296	344	397	366		433	482
PentaD					122	120	140		240	224
THO1	152	133	175	543	607	476	660	231	493	585
vWA		124	233	434	295	290	694	291	781	755
			120	327	300	284	674	187	740	591
D21			161	458	208	194	447	238	511	650
			116	385	188	201	491	155	489	581
D7				187	164		228	126	311	291
				165			241		304	280
D5				344		129	291	158	424	380
				257			269	161	510	429
TPOX				278	258	339	290	153	236	246
DYS391				128			144		175	305
D8	238	564	449	872	459	713	1229	589	1977	1874
	114	480	378	1066	516	812	1465	716	2079	1302
D12	399	292	759	1126	485	573	1086	857	1927	1068
		217	618	986	441	644	839	1175	1918	1300
D19		135	139	525	540	466	842	388	1358	777
		122	135	516	307	492	707	387	1064	864
FGA			157	564	246	210	478	454	804	989
			158	331	173	169	531	181	907	708
D22				183	217	129	163		171	199
				142	208				149	166

**Table 4:** **DNA Profiles from DFP Indirect Transfers.** For each of the ten replicate profiles generated from DNA collected from the knife sheath snaps, peak heights (RFU) are listed. DNA quantification results and the amount of DNA added to each PCR are also included**.** An empty box indicates that no allele above the analytical threshold of 110 rfu was detected.

DFP: Indirect	DI1	DI2	DI3	DI4	DI5	DI6	DI7	DI8	DI9	DI10
Quant (ng/μl)	0.017	0.014	0.012	0.015	0.010	0.011	0.006	0.0059	0.0097	0.0097
Total DNA (ng)	0.52	0.43	0.35	0.44	0.31	0.32	0.19	0.18	0.29	0.29
Added to PCR (ng)	0.26	0.21	0.18	0.22	0.15	0.16	0.093	0.089	0.15	0.15

	rfu	rfu	rfu	rfu	rfu	rfu	rfu	rfu	rfu	rfu
Amel	272	121	258	309	169	170	434	313	557	349
									165	142
D3	193					182	122	164	128	122
						123		295	157	
D1			135	135	206		123		161	131
							130		137	138
									214	190
D2S441	254		203	145			177		186	129
D10	139		119						214	121
D13	116								288	
PentaE					159		126			
					100					
D16							115	124	144	
									167	142
D18								117	168	
D2								120		
CSF										
PentaD										
THO1								112	120	128
vWA									154	
									121	
D21									170	
									151	
D7										
D5								116	112	160
									117	
TPOX										
DYS391										
D8			306	234	207	135	117	254	477	371
			401				145		241	282
									157	139
D12			116					111	110	135
								173	123	116
									312	208
D19	118					138	294	262		405
	113									
FGA		156		119			121		119	
							177			
							204			
D22			119		117					

For the additional DFP extracts from direct transfer snaps that were used in profiling, the mean total DNA recovered was 0.79 ng, SD 0.49 (5.3% of the DNA deposited), and the mean POI DNA recovered was 0.52 ng, SD 0.34 (5.8% of the POI DNA deposited). Although only the male POI DNA was added to the direct transfer, low levels of female DNA were also detected in all ten samples (mean = 0.28 ng). Prior to completing the DNA transfers, we confirmed that the surfaces were free from DNA, therefore, the domesticated POI hand introduced a low quantity of background female DNA, similar to real-life where exogenous DNA is picked up during normal activity. The female-originating alleles were not detectable in the DNA profiles (>110 rfu, or >20% in the stutter position). The sample set produced three full DNA profiles, three with allele drop-out at one locus, two with drop-out at 2 loci, and the final two profiles with drop-out at 9 and 18 loci (Table 3).

The DFP indirect extracts remained intact, and a subset of ten indirect snap extracts were used for DNA profiling. For this subset, the mean POI DNA was 0.15 ng, SD 0.08 (1.7% of the POI DNA deposited). The mean total DNA was 0.35 ng, SD 0.11 (2.3% of the total DNA deposited), and the mean OP DNA was 0.20 ng (3.3% of the OP DNA deposited). No SD was determined for this estimated value. Five of the profiles contained too few alleles above the analytical threshold (110 rfu) to conduct an analysis. In the remaining five profiles, mixture analysis determined the POI:OP ratios to be: 38:62 (Replicate 1); 32:68 (R7); 26:74 (R8); 42:58 (R9); 39:61 (R10), with an average peak height ratio of 1:1.6 (POI:OP) (Table 4).

For the WFP direct samples, the mean POI DNA was 2.2 ng (SD 1.7), range 0.91 - 6.6 ng. The mean total DNA was 2.9 ng (SD 2.2), range 1.1 – 8.9 ng. Although there was a single male donor in the direct transfers, we detected a low-level background of female DNA in all ten of the samples; the mean female DNA was 0.77 ng, range 0.195 – 2.4 ng (no SD determined for this calculated value). We confirmed that the surface was decontaminated prior to the experiments, therefore the male hand introduced a low quantity of background female DNA, consistent with real-life. However, the female-originating alleles were not detectable in the DNA profiles (>110 rfu, or >15% in the stutter position). The direct sample set from wild fingerprints produced 9 full POI profiles and 1 POI profile missing both alleles at 6 loci (Table 5).

**Table 5:** **DNA Profiles from WFP Direct Transfers.** For each of 10 replicate profiles generated from DNA collected from the knife sheath snaps, the peak heights, in rfu, are listed. The DNA quantification results and quantity of DNA added to each PCR are included. An empty box indicates that no allele above the analytical threshold of 110 rfu was detected.

WFP: Direct	WD1	WD2	WD3	WD4	WD5	WD6	WD7	WD8	WD9	WD10
Quant (ng/μl)	0.037	0.061	0.057	0.088	0.11	0.084	0.072	0.06	0.12	0.30
Total DNA (ng)	1.1	1.8	1.7	2.6	3.2	2.5	2.2	1.8	3.5	8.9
Added to PCR (ng)	0.50	0.50	0.50	0.50	0.50	0.50	0.50	0.50	0.50	0.50

	rfu	rfu	rfu	rfu	rfu	rfu	rfu	rfu	rfu	rfu
Amel	1017	2417	872	751	1713	1792	884	527	2057	2413
	1180	1929	839	568	2363	1260	1154	690	2147	2208
D3	1090	1539	1005	647	1470	1271	827	411	1776	2245
	1009	1697	750	565	1071	1050	757	480	2251	1768
D1	972	1609	548	514	949	1319	797	669	1375	2462
	591	1326	612	471	692	1478	400	486	1719	2099
D2S441	658	1350	311	144	930	1024	561	402	937	1347
	514	1330	372	438	829	740	494	431	753	1795
D10	570	1157	307	282	762	1162	554	418	947	1666
	727	789	256	294	521	566	477	355	1020	1258
D13	688	1571	279	345	1266	1121	570	523	1189	2430
PentaE	458	538		151	572	354	268	275	608	865
	273	491			464	379	243	189	340	835
D16	867	1122	708	374	690	825	696	419	1091	1586
	924	946	687	435	553	936	541	508	911	1334
D18	283	645	282	210	251	360	227	174	522	708
	246	450	165	114	230	327	107	185	280	725
D2	1059	1050	342	453	721	980	395	437	1247	2266
CSF	521	639	193	228	598	919	257	302	823	1449
PentaD	176	280		116	340	263	145	248	388	631
THO1	1123	1341	1306	431	692	1092	823	625	1336	1538
vWA	403	709	320	299	459	681	426	332	606	1076
	293	849	216	256	521	559	344	258	548	1207
D21	443	813	413	313	484	592	398	338	484	911
	444	492	287	273	276	573	271	219	909	793
D7	277	413		130	190	424	170	138	498	614
	318	403		176	170	268	188	133	365	569
D5	184	360		235	254	367	235	191	218	685
	112	423		270	372	448	221	246	396	611
TPOX	431	730		396	432	591	381	302	591	1297
DYS391	113	375		161	176	530			397	588
D8	981	1304	953	805	1434	1707	1586	1149	2452	2985
	1348	2555	705	766	1266	2047	1032	736	2503	3207
D12	924	1272	702	796	675	1607	1182	538	1315	1795
	937	1306	1330	976	668	1813	953	511	1132	1478
D19	1036	1649	421	708	870	1340	772	259	1113	1546
	1146	1346	514	673	767	1408	450	470	1473	1999
FGA	405	1064	167	324	496	567	354	457	889	1411
	266	541	127	420	587	552	334	518	805	1607
D22	265	671		242	398	455	274	118	415	1040
	146	310		296	196	642	291		319	1098

For the 10 indirect WFPs, the mean POI DNA was 0.15 ng (SD 0.12), range 0.0 – 0.34 ng. The mean total DNA was 0.83 ng (SD 0.61), range 0.0 – 1.97 ng. The mean OP DNA was 0.68 ng, range 0.0 – 1.8 ng (no SD determined for this estimated value). Five of the samples showed no alleles above the analytical threshold (110 rfu). The remaining five contained POI:OP mixtures as follows: 14:86 (Replicate 1); 51:49 (R4); 27:73 (R7); 17:83 (R8); and 27:72 (R9), with an average peak height ratio of 1:2.7(POI:OP) (Table 6).

**Table 6:** **DNA Profiles from WFP Indirect Transfers.** For each of the ten replicate profiles generated from DNA collected from the knife sheath snaps, peak heights (RFU) are reported. DNA quantification results and the amount of DNA added to each PCR are also included. In sample WI7, one drop-in allele is noted at the D12 locus. An empty box indicates that no allele above the analytical threshold of 110 rfu was detected.

WFP: Indirect	WI1	WI2	WI3	WI4	WI5	WI6	WI7	WI8	WI9	WI10
Quant (ng/μl)	0.027	0.039	0.021	0.020	0.029	0	0.050	0.024	0.066	0
Total DNA (ng)	0.81	1.2	0.64	0.61	0.86	0	1.5	0.72	2.0	0
Added to PCR (ng)	0.41	0.59	0.32	0.30	0.43	0	0.74	0.36	0.98	0

	rfu	rfu	rfu	rfu	rfu	rfu	rfu	rfu	rfu	rfu
Amel	1000			884			2027	1295	2373	
				338			208	197		
D3	419			179			953	730	903	
	592			327			858	838	984	
				496			218	176	177	
D1	341			283			734	543	533	
	315			215			252	322	144	
				297			537		126	
				113					514	
D2S441	236			203			452	230	412	
	259						398	297	689	
D10	451			223			604	358	857	
D13	143			111			288	167	472	
							284	298	254	
							164			
PentaE							178		201	
							113		273	
D16	292			245			294	549	470	
	171			369			684	338	453	
				129			522			
D18	105			135			326	167	325	
	137						271	132	334	
D2				305			289		305	
							313		146	
CSF							238		184	
									112	
PentaD							114		138	
									143	
THO1	229			152			204	337	497	
	146			393			359	330	317	
vWA	130						322	291	124	
	171						285	227	449	
									465	
D21	125			134			324	169	311	
							155		433	
D7	162						319	123	374	
D5				177			285		216	
							230		320	
TPOX	166			152			258		236	
DYS391										
D8	879			623			1054	1121	1258	
	577			467			910	1303	1390	
				927			450	143	311	
				405			313		228	
D12	121			195			299	195	318	
	528			442			130	313	1035	
	343			471			581	896	666	
				528			995	898		
							653			
D19	143			179			595	448	745	
	334			343			1182	547	675	
				169						
FGA	190			131			252	176	110	
	135			120			261		663	
							118		502	
D22	124						128	149	267	
									190	

### 3.5 Transfer probabilities

The results of our experiments can be summarized as transfer probability tables using extrinsic characteristics of the DNA profiles, that is, features associated with the profiles that are independent of the DNA sequence itself [33]. We report the data given one of two propositions: a) Kohberger, the POI, touched the knife sheath; or b) Kohberger shook hands with some other person (OP) who touched the knife sheath. We define five possible outcomes:

1) no detectable transfer of POI DNA: there was no profile, or the POI (or OP) alleles were not included in the profile no obligate alleles of the POI observed: this is the event T_0
2) detectable transfer, mixed sample with approximately equal contributions from POI + OP, ±10% or 45:55; this is the event T_NoMajor&Minor.
3) detectable transfer, mixed sample in which POI is the major (>55%); this is the event T_Major
4) detectable transfer, mixed samples in which POI is the minor (<45%); this is the event T_Minor
5) detectable transfer, single-source in which POI is the only contributor (whether full or partial); this is the event T_SingleSource

The transfer probabilities generated by DNA profiles from domesticated fingerprints are summarized in Table 3, given that the POI, the alleged perpetrator, contacted the knife sheath snap, in: A) a direct transfer; B) an indirect transfer. We assign uniform prior counts and define transfer probability = [(observed count) +(prior count)]/[(total observed + total prior counts)], and, to avoid division by zero, posterior counts = prior counts + observed counts.

Given that the POI contacted the sheath snap in a direct transfer (domesticated fingerprints, Table 7), the transfer probability for a single-source POI DNA profile is 0.50 (Table 7A); this is reduced to 0.067 if the transfer is indirect (Table 7B).

**Table 7:** Transfer Probabilities, Domesticated Fingerprints. Transfer probabilities were calculated from DNA profile data in: A) a direct transfer and B) an indirect transfer.

A) Domesticated Fingerprints: Direct Transfer				
Event	Prior Counts	Observed Counts	Posterior Counts	Transfer Probability
T_0	1	0	1	0.125
T_NoMajor&Minor	1	0	1	0.125
T_Major	1	0	1	0.125
T_Minor	1	0	1	0.125
T_SingleSource	1	3	4	0.50
Total	5	3	8	1.0

B) Domesticated Fingerprints: Indirect Transfer				
Event	Prior Counts	Observed Counts	Posterior Counts	Transfer Probability
T_0	1	5	6	0.40
T_NoMajor&Minor	1	0	1	0.067
T_Major	1	1	2	0.13
T_Minor	1	4	5	0.33
T_SingleSource	1	0	1	0.067
Total	5	10	15	1.0

After an indirect transfer (domesticated fingerprints, Table 7B), there is a 40% probability that there will be no detectable DNA profile. There is a 53% probability of producing a mixed profile, most likely with a POI minor donor/OP major donor (33%). The probability of observing a POI single-donor profile is unlikely, only 6.7%.

Table 8 presents the DNA profiling results for wild fingerprint samples, assuming the POI DNA was: A) a direct transfer and B) an indirect transfer. The transfer probabilities calculated for WFP direct transfers are identical to those obtained for domesticated fingerprints. The transfer probabilities for indirect transfers calculated for the WFPs match those for the DFPs, except that the values for an approximately equal POI:OP mixture and for a mixed sample with a major POI component are reversed, indicating that there is a slightly higher probability of finding an equivalent contribution from POI and OP in wild fingerprints.

**Table 8:** Transfer Probability Table, Wild Fingerprints Transfer probabilities were calculated from DNA profile data in: A) a direct transfer and B) an indirect transfer.

A) Wild Fingerprints: Direct Transfer				
Event	Prior Counts	Observed Counts	Posterior Counts	Transfer Probability
T_0	1	0	1	0.125
T_NoMajor&Minor	1	0	1	0.125
T_Major	1	0	1	0.125
T_Minor	1	0	1	0.125
T_SingleSource	1	3	4	0.50
Total	5	3	8	1.0

B) Wild Fingerprints: Indirect Transfer				
Event	Prior Counts	Observed Counts	Posterior Counts	Transfer Probability
T_0	1	5	6	0.40
T_NoMajor&Minor	1	1	2	0.13
T_Major	1	0	1	0.067
T_Minor	1	4	5	0.33
T_SingleSource	1	0	1	0.067
Total	5	10	15	1.0

### 3.6 Domesticated versus wild fingerprints in DNA transfer pathways

The empirical data presented here shows that the information provided by transfer analysis of DNA profiling data can factor into activity-level propositions. We have demonstrated that the domesticated trace DNA pathway is a conservative surrogate for the wild trace DNA pathway. Note the robustness of the domesticated study with its relatively small SD values with respect to the wild study with its comparatively larger SD values, which are greater than the mean values. These contrasting studies demonstrate the value of the model domesticated approach to perform replicates to examine specific steps of the overall activity and analysis process in controlled circumstances. In comparison, the wild study shows much more variation relative to the domesticated, which conforms to casework experience where more variation is expected.

Any time a model is produced, there are distinct differences between the model and the real world necessitated by assumptions built into the experimental design. However, as long as the model reasonably approximates real life, tremendous value can be gleaned through the capability to hold all but one variable constant, to determine the true impact of that single variable on the experiment outcome. It is of significant value that the model is conservative in that more DNA was transferred from the previous transfer, highlighting the maximum potential for indirect transfer. This may be due to the relative smoothness of the domesticated hand relative to real hands, particularly at the microscopic level. There may be other factors, such as surface charge and chemistry which may increasingly hold previously transferred cells in place in the wild, which favors the latest transfer as a greater component in real life versus the domesticated model. This will be a feature of future experiments now enabled to further test the model and even better hone its features to match those of the wild.

### 3.7 Answering activity-level questions

In the direct transfer pathways (Proposition 1), for both domesticated and wild fingerprints, only the POI DNA profile was detectable above the analytical threshold. In the indirect transfer pathways (Proposition 2), using both the domesticated and wild fingerprints, four out of the five.

DNA profiles generated from the knife sheath snap contained a majority contribution from the OP, the final handler.

### 3.8 Theories of trace DNA evidence

Grounded in the empirical results of the present study and consistent with findings reported in the literature, our results give rise to six Theories of Trace DNA Evidence [21,34,[37], [38], [39], [40]]: 1) Every Contact Establishes a New Equilibrium; 2) Diminishing Returns, 3) Majority Contributor is more (likely) from the Last Handler; 4) DNA is not a Glitter Bomb 5) Primordial Soup Mix; and 6) DNA is not self-cleaning. These theories are derived from, and should be interpreted within, the experimental conditions and transfer pathways examined in this study; future studies will determine if alternative mechanisms of transfer agree with these observations. The first three theories are discussed above (Section 2.2). Theory number four states that DNA is not a glitter bomb, it does not become airborne and manifest itself universally in large analyzable quantities without a transfer mechanism. The subject hands in this study, both domesticated and wild, are a transfer mechanism that are real and cannot be fully sterilized, hence a small quantity of extraneous DNA can be introduced with the cells of the DFP, or the wild hand, as demonstrated by our results. We did not observe a glitter bomb-like explosion of large quantity and quality of un-trackable DNA that disrupted the surface-to-surface transfer mechanism as we have seen in our experiments. In fact, the trace quantity demonstrates a realistic scenario and also quantifies and qualifies the degree of extraneous DNA that is randomly carried about by transfer vectors - it is not zero, but it is not a glitter bomb of surprise profiles that disrupt evaluation.

The final two theories arise from the idea that DNA found on surfaces in the environment will rarely be single source. This was evident in our experiments as we needed to implement extensive decontamination procedures to ensure background-free surfaces. At a crime scene, the surfaces are complex, they are a “primordial soup” mixture of persisting DNAs and various forms of cellular debris (Trace DNA Theory #5). Trace DNA Theory follows the laws of entropy and thermodynamics, where objects move from a higher energy state (single source DNA) to a lower energy state (DNA mixtures). Single source DNA is relatively rare and takes energy to maintain; when DNA leaves the body as single source it begins to contact other DNA, thereby becoming a DNA mixture which becomes increasingly complex with additional steps. Once DNA becomes part of this complex primordial soup mixture, a single DNA cannot be physically separated from the mixture and revert to single-source status without human intervention (Trace DNA Theory #6). As an inanimate object, DNA does not move towards single source from a mixture, unless more of one type of DNA is added until the rest of the DNA is reduced so as to become a diminishing return that is no longer detected, or greatly in the minority contribution.

The real-life crime scene is complex and necessitates the analysis of experimental pathways, like the ones we describe to represent the best-case scenarios that yield the most DNA and the best DNA profiles we can expect. The finder-of-fact faces a daunting challenge in weighing alternative scenarios to explain crime scene DNA evidence. Better data means better interpretations. Forensic scientists’ education, training, and experience, coupled with the reproducible ground truth data provided here can provide opinion evidence to assist the finder-of-fact to arrive at better, more data-grounded justice.

## 4 Conclusions

During the investigation of the November 13, 2022 homicides in Moscow, Idaho, a tan leather knife sheath was recovered from a bed next to one of the deceased victims. Analysis of the sheath yielded a single-source male DNA profile from biological material collected from the button snap closure [41]. An initial investigative comparison using a reference sample obtained from discarded trash associated with the defendant’s family residence could not exclude a biological father–son relationship, with 99.9998% of the male population expected to be excluded [41]. Following arrest, a direct reference sample was obtained and compared using autosomal STR analysis [42]. The evidentiary findings were evaluated within a likelihood ratio framework under competing source-level propositions (H_1_: the defendant is the source; H_2_: an unknown, unrelated individual is the source), producing a likelihood ratio on the order of 10^27^, indicating that the DNA results were many orders of magnitude more probable if the defendant were the source than if an unrelated individual were the source [43]. The sheath DNA therefore constituted the central source-level forensic evidence linking the defendant to the crime scene. In July 2025, the defendant pleaded guilty in Idaho state court to four counts of first-degree murder and one count of felony burglary pursuant to a plea agreement in which the state withdrew its pursuit of the death penalty; he was subsequently sentenced to four consecutive terms of life imprisonment without the possibility of parole and an additional fixed term for the burglary conviction [44].

Source-level likelihood ratios address donor attribution but do not resolve activity-level propositions concerning the mechanism, timing, or circumstances of biological material deposition [45,46]. The detection of DNA on a handled object may be compatible with multiple transfer pathways, including direct contact, secondary transfer, and background contributions, the probabilities of which depend on substrate, contact dynamics, and persistence [25]. Despite increasing recognition of the importance of activity-level evaluation, empirical datasets capable of informing such assessments remain comparatively limited. The experimental probability data generated in the present study—including quantification of DNA recovery following controlled direct and indirect transfer events—provide proposition-relevant information suitable for incorporation into activity-level likelihood assessments.

With the data presented here, we have demonstrated that the domesticated trace DNA pathway is a conservative surrogate for the wild trace DNA pathway. The similarity in the total DNA deposited by DFPs (domesticated fingerprints) and WFPs (wild fingerprints) and the relative ratios of male/female confirm that the domesticated pathway is a conservative surrogate for the wild pathway. The wild pathway shows greater variation as seen through larger standard deviation.

We designed the domesticated fingerprint/hand model to provide conservative estimates of DNA loss/recovery compared to real-life scenarios, including those of the University of Idaho 4X homicide with POI (person of interest) Bryan C. Kohberger. Kohberger’s DNA was found on a knife sheath at the crime scene. Experiments were run under pristine laboratory conditions, with no effects of detrimental environmental factors. Transfers were made by full contact hand-to-hand with no time lapse between fingerprint deposition and transfer. These are the most “direct,” indirect routes by which these transfers could occur; therefore, they provide more ideal scenarios favoring indirect transfer than real life.

These results confirm that there is a wide range of DNA recoveries after both direct and indirect pathways, which is expected with wild trace samples. With such a wide range of starting quantities of DNA deposited in a wild fingerprint, it is not possible to reasonably assign a starting value that could be used in empirical calculations. Therefore, it is necessary to employ a ground truth sample to generate empirical data in realistic-scenario, activity studies that support evaluative, activity-level reporting.

In the direct transfer pathways (Proposition 1), for both domesticated and wild fingerprints, only the POI DNA profile was detectable above the analytical threshold. In the indirect transfer pathways (Proposition 2), using both the domesticated and wild fingerprints, four of the five typeable DNA profiles generated from the knife sheath snap contained a contribution greater than 55% from the OP (other person), the final handler. Therefore, based in empirical data, we can state that a POI major DNA profile is more likely if a direct transfer occurred rather than an indirect transfer.

These results support six Theories of Trace DNA Evidence: 1) Every Contact Establishes a New Equilibrium; 2) Diminishing Returns, 3) Majority Contributor is more (likely) from the Last Handler; 4) DNA is not a Glitter Bomb 5) Primordial Soup Mix; and 6) DNA is not self-cleaning. With the six theories and data produced from these studies, the forensic scientist is in a better position to provide expert opinion evidence to support decisions to be made by the finder of fact in criminal trials.

## Data availability statement

The datasets generated during and/or analyzed during the current study are being prepared for submission to the National Archive of Criminal Justice Data (NACJD, https://www.icpsr.umich.edu/web/pages/NACJD/archiving/deposit-nij-ojjdp.html). The datasets are also available from the corresponding author on reasonable request.

## CRediT authorship contribution statement

**Aldrin Alviar:** Conceptualization, Data curation, Formal analysis, Methodology, Validation, Visualization, Writing – original draft, Writing – review & editing. **Ray Wickenheiser:** Conceptualization, Formal analysis, Methodology, Visualization, Writing – original draft, Writing – review & editing. **Ashley Hall:** Conceptualization, Data curation, Formal analysis, Funding acquisition, Project administration, Supervision, Visualization, Writing – original draft, Writing – review & editing.

## Declaration of competing interest

The authors declare that they have no known competing financial interests or personal relationships that could have appeared to influence the work reported in this paper.
